# Norrin in cancer: from a promising prognostic biomarker to a novel therapeutic target

**DOI:** 10.3389/fonc.2025.1692715

**Published:** 2025-11-21

**Authors:** Jiaqing Zhang, Zhengyi Zhang, Huabao Xiong, Guanjun Dong, Lu Yu

**Affiliations:** 1 Institute of Immunology and Molecular Medicine, Jining Medical University, Shandong, China; 2 Jining Key Laboratory of Immunology, Jining Medical University, Shandong, China

**Keywords:** anticancer, cancer hallmarks, tumor microenvironment, Norrin, Wnt signaling

## Abstract

Cancer continues to pose significant risks to public health globally due to incomplete therapeutic conquests even though significant advances have been achieved in the field of oncology. Therefore, understanding the molecular mechanisms driving tumorigenesis and progression is critical for developing novel treatment strategies to achieve effective cancer treatment. Norrin, the secreted cystine-knot protein originally recognized for its functional role in retinal vascular development an neuronal protection, is now implicated in oncogenic processes. This review synthesizes the existing evidence on Norrin’s involvement in tumors, highlighting its aberrant expression across multiple malignancies and its functional role in cancer cell proliferation, migration, invasion, and tumor-associated angiogenesis. The compelling data reported in this review suggest that dysregulated Norrin signaling promotes oncogenesis in various cancer types. Furthermore, the mechanistic basis of Norrin’s tumorigenic effect is discussed, and the therapeutic potential of targeting Norrin is evaluated to provide novel insights for future diagnostic and therapeutic development in oncology.

## 1 Introduction

The cystine-knot protein Norrin (*NDP*), originally identified as the Norrie disease protein, is a secreted factor that reportedly promotes vascular growth in the retina and exhibits neuroprotective activity (1). *NDP* serves as a non-canonical ligand for the FD4R. The pathogenic variants in the *NDP* gene lead to Norrie disease, a genetic disorder primarily manifesting as abnormal retinal development and severe vision impairment beginning from birth or early infancy (2). The missense mutation of the *NDP* gene can trigger familial exudative vitreoretinopathy (FEVR) (3, 4). Pathogenic variants in the *NDP* gene have been associated with multiple ocular pathologies, among which the notable ones are Coats disease and retinopathy of prematurity (5, 6).

Malignancies continue to pose a significant health burden worldwide, ranking as the second most common cause of death globally. Notably, malignancies are the predominant cause of death in people aged less than 85 years (7, 8). According to the International Agency for Research on Cancer (IARC), the global cancer incidence reached about 20 million newly diagnosed cases in 2022, with projections suggesting that this number may rise to 35 million by 2050. Notably, lung cancer has the highest incidence among all cancer types, accounting for 12.4% of cases (9, 10). The existing treatment modalities mainly include traditional radiotherapy, surgery, and chemotherapy, while the emerging technologies, such as nanomedicine, are also being investigated (11, 12). Nevertheless, all these treatment approaches have drawbacks, including high medical expenses, severe adverse reactions in patients, and poor treatment outcomes (13–15). Consequently, research to identify novel therapeutic targets and treatment strategies for cancer is necessary. Growing experimental evidences support Norrin’s functional significance in multiple neoplastic processes, spanning adenocarcinomas of the digestive tract, lung carcinomas, and neurological tumors (16–19). highlighting directions for future research. Clarifying these mechanisms will help uncover Norrin’s potential as a therapeutic target in oncology and facilitate its clinical translation, thereby offering new strategies for cancer treatment.

## 2 The gene locus and expression levels of Norrin

**Figure 1:**
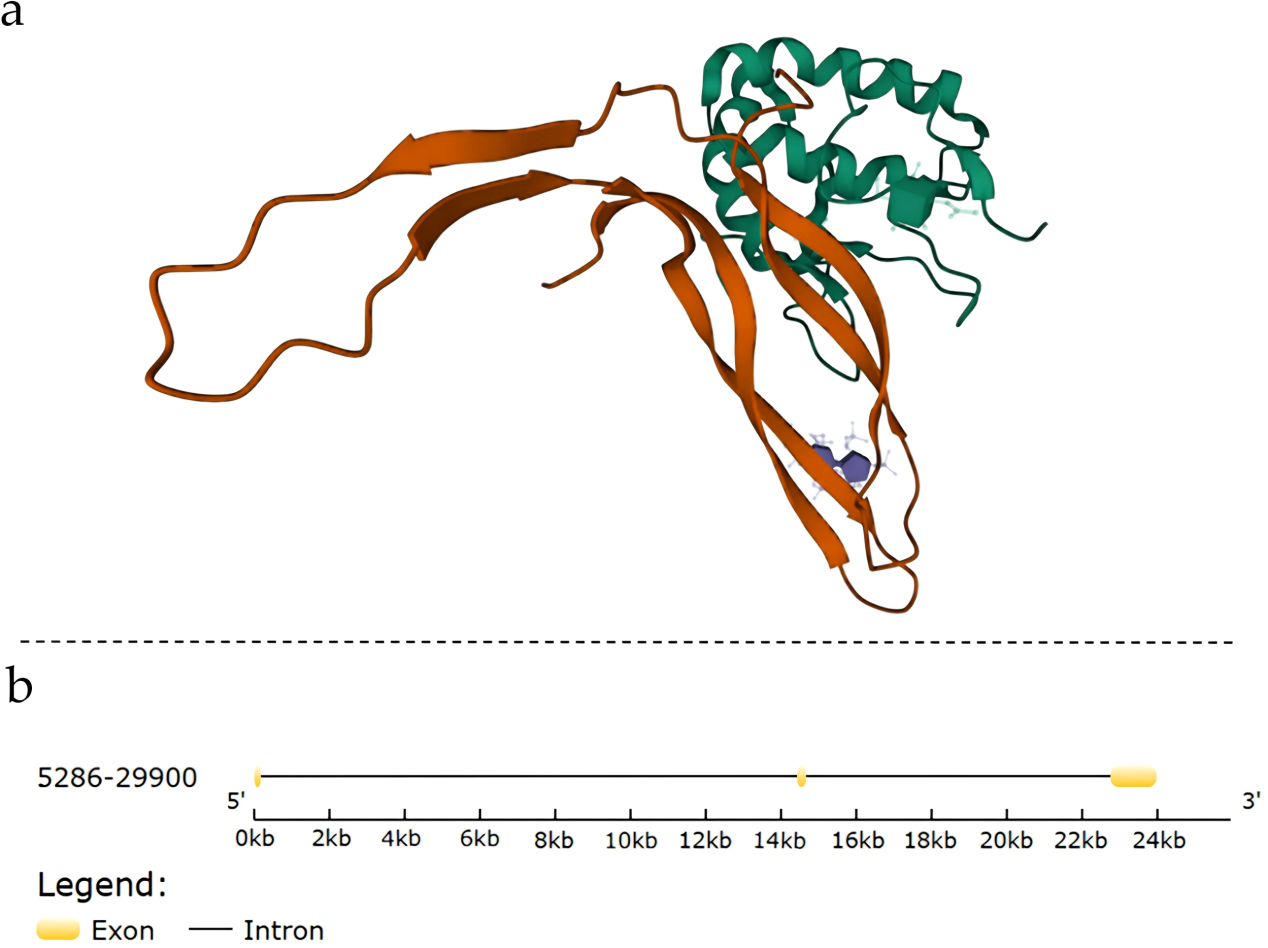
Structural and genomic information of Norrin: **(A)** Complex structure of Norrin; **(B)** Gene structure of Norrin. Exons are indicated using yellow boxes, and introns are indicated using the connected black lines.

The *NDP* gene is located on the X chromosome(Xp11.4)and comprises three exons, encoding Norrin, a protein composed of 133 amino acids (**Figure 1**) (20).

The Human Protein Atlas has revealed that *NDP* is expressed in various tissues, mainly in the brain, eyes, reproductive, muscle, and soft tissues (21). The integrated analysis of the Cancer Cell Line Encyclopedia (CCLE) and The Cancer Genome Atlas (TCGA) datasets revealed ubiquitous *NDP* expression across multiple malignancies, with the highest expression levels observed in the glioma cell lines and primary glioblastoma specimens (22, 23). The cellular level expression of *NDP* is increased in gastric cancer parenchymal cells, but it remains undetected or has the lowest expression in endothelial cells (18). Similar to its differential expression across various tissues, Norrin has variable intracellular expression levels and is relatively highly expressed in the plasma membrane and nucleus (https://www.genecards.org/) (24).

## 3 The role of norrin in normal cells

Norrin is a secreted cystine-knot growth factor initially identified for its critical role in neuroprotective processes (2, 6, 25). Its physiological roles in normal tissues and cells throughout the body are primarily mediated through angiogenesis and neuroprotection, with other functions playing secondary roles. It is also essential for angiogenesis, contributing to the development, maintenance, and remodeling of the retinal vasculature.

Norrin also modulates vascular growth and organization during ocular development and in mature vascular networks. Moreover, Norrin prevents, to a large extent, hyperoxia-induced vascular damage (2, 6, 20). Interestingly, during embryonic development, Norrin is important for both neuroprotection and the regulation of angiogenesis, processes essential for embryonic vascular development and retinal neuron growth (26). In tissues other than the eyes, Norrin helps maintain the blood-retinal and blood-brain barriers, regulates angiogenesis in the cochlea and uterus, and maintains the cerebellar vascular system (27–30). Furthermore, studies have demonstrated that Norrin promotes cardiomyocyte differentiation by enhancing the induction of the cardiac progenitor cells derived from pluripotent stem cells (31).

**Figure 2:**
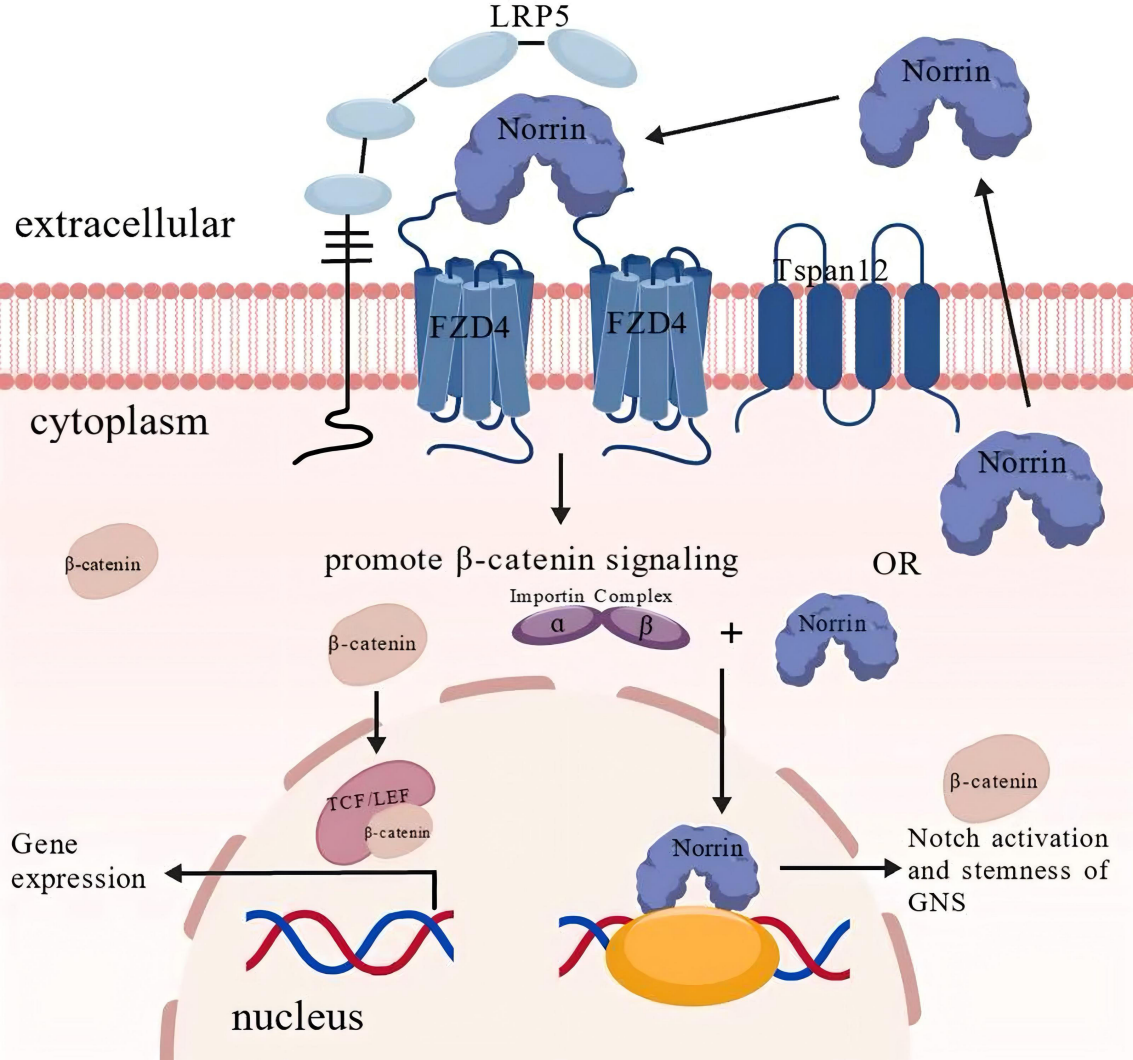
The normal physiological function and mechanism of Norrin. Under physiological conditions, Norrin serves as an endogenous ligand for FZD4, activating canonical Wnt/β-catenin signaling through receptor binding. Upon receptor stimulation, β-catenin accumulation and nuclear translocation occur, following which β-catenin associates with the TCF/LEF transcription factors, forming a functional complex that modulates downstream gene expression. Notably, emerging evidence suggests that Norrin may also perform β-catenin-independent functions through alternative signaling pathways. Importin-α mediates the nuclear translocation of Norrin, which enhances glioblastoma neural stem (GNS) cell proliferation and potentiates Notch signaling activation. This figure was created using BioGDP.com (33).

In normal cells, Norrin functions as a ligand, and its β-sheet structurally mimics the Wnt finger loop, enabling its binding to FZD4. Upon recruitment of the coreceptor LRP5 and the auxiliary protein TSPAN12, the ternary complex formed by Norrin, FZD4, and LRP5 initiates β-catenin signaling, resulting in cytoplasmic β-catenin accumulation (32). Subsequently, the β-catenin is translocated to the nucleus, where it interacts with the TCF/LEF transcription factors, initiating and maintaining the transcriptional activation of target genes (**Figure 2**) (34–36).

## 4 The role of Norrin in cancer cells

**Figure 3:**
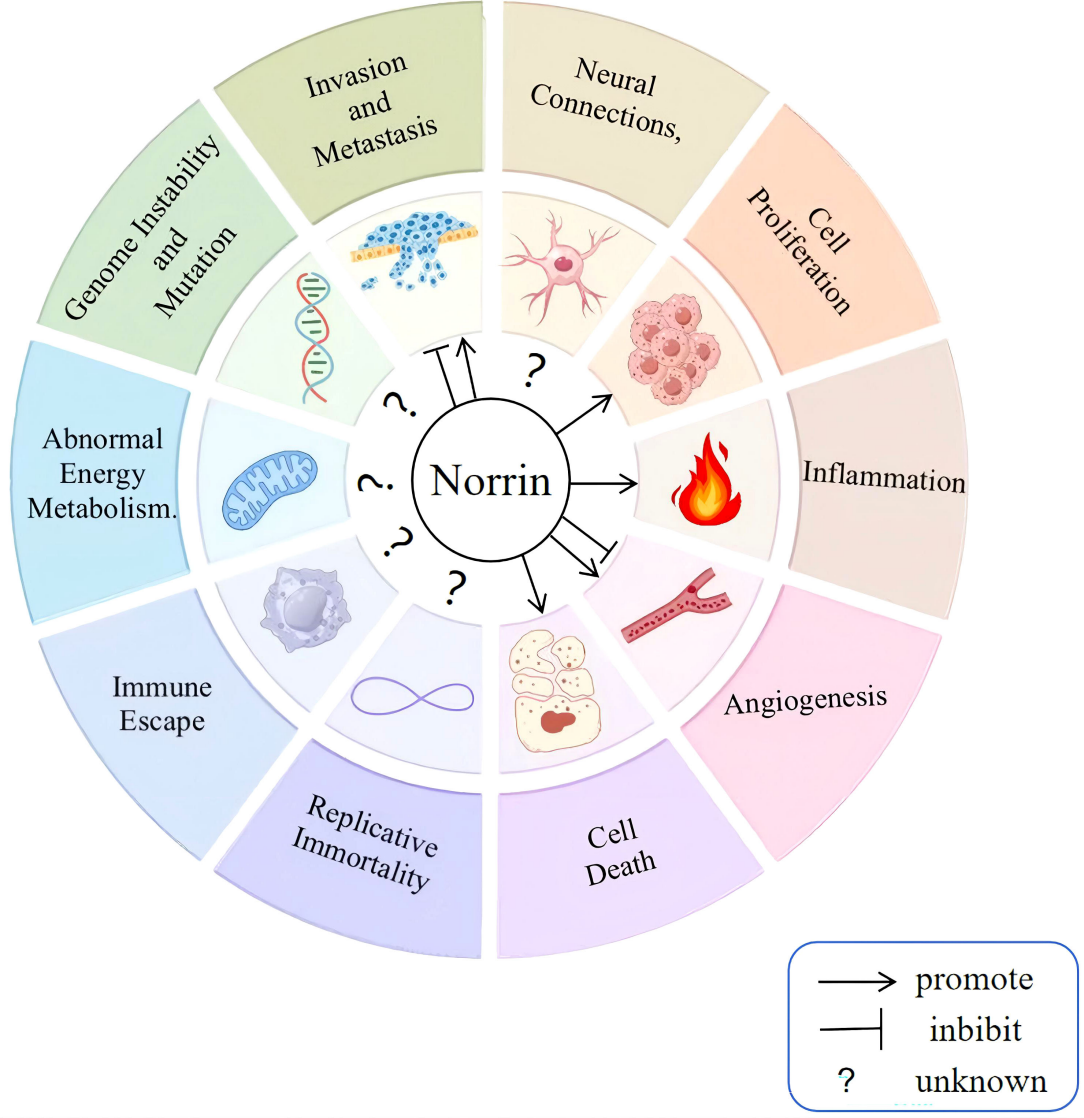
Effect of Norrin on cancer progression. The defining features of cancer include dysregulation of the tumor microenvironment, altered metabolic pathways, genomic instability, enhanced migratory capacity, persistent growth signaling, pro-inflammatory responses, neovascularization, evasion of apoptosis, unlimited replicative potential, and immune escape mechanisms. Norrin has been confirmed to promote sustained proliferation, resistance to cell death, and inflammation in cancer cells while exerting both promoting and inhibitory effects on migration, invasion, and angiogenesis. However, no study has demonstrated its effect on the remaining five hallmarks.

Ten distinct biological capabilities, to date, have been established as fundamental hallmarks of cancer (37, 38), and building upon these defining hallmarks of cancer, this article elaborates on how Norrin affects cancer to provide an in-depth understanding of its potential role in the pathways leading to the development of malignancy in cancer (**Figure 3**). Norrin is dysregulated across multiple malignancies and contributes to tumorigenesis and cancer progression through specific mechanisms (**Table 1**) (16–19, 40).

**Table 1:** The role of Norrin on the hallmarks in different cancer cell lines.

Cancer type	Hallmarks of cancer	Cancer cell line	Effect	Reference
Gastric cancer	Proliferation	SGC7901, MGC803, AGS, XN0422	Upregulate	(16)
Glioblastoma	Proliferation	G523, G472, G440	Upregulate	(38)
Glioblastoma	Proliferation	G411, G564	Downregulate	(38)
Medulloblastoma	Proliferation	GNP, mMF	Downregulate	(39)
Gastric cancer	Invasion and Metastasis	SGC7901, MGC803, AGS, XN0422	Upregulate	(16)
Lung cancer	Invasion and Metastasis	A549, H1299	Upregulate	(18)
Colon cancer	Angiogenesis	CaCO2	Upregulate	(17)

The mechanism of action of Norrin in tumors is highly context-dependent, primarily determined by the specific signaling pathways and cellular microenvironment. For example, Norrin exerts dual effects on glioma stem cells (GSCs) in an ASCL1-dependent manner. Glioma Stem Cells (GSCs) are a small subpopulation of self-renewing cells within gliomas. In ASCL1^lo^ GSCs, it suppresses proliferation by activating the FZD4-mediated Wnt/β-catenin cascade. Conversely, in ASCL1^hi^ GSCs, it promotes tumor progression through a Wnt-independent activation of the Notch signaling pathway (40, 41). Worthwhile, it exhibits a bidirectional nature that depends on the cellular context. Genetic ablation of Norrin in gastric cancer cells significantly impairs their invasive capacity (16). Conversely, in pulmonary carcinoma models, elevated Norrin expression activates the Wnt pathway and similarly enhances the migratory and invasive capacities of A549 and H1299 cells (18).

**Figure 4:**
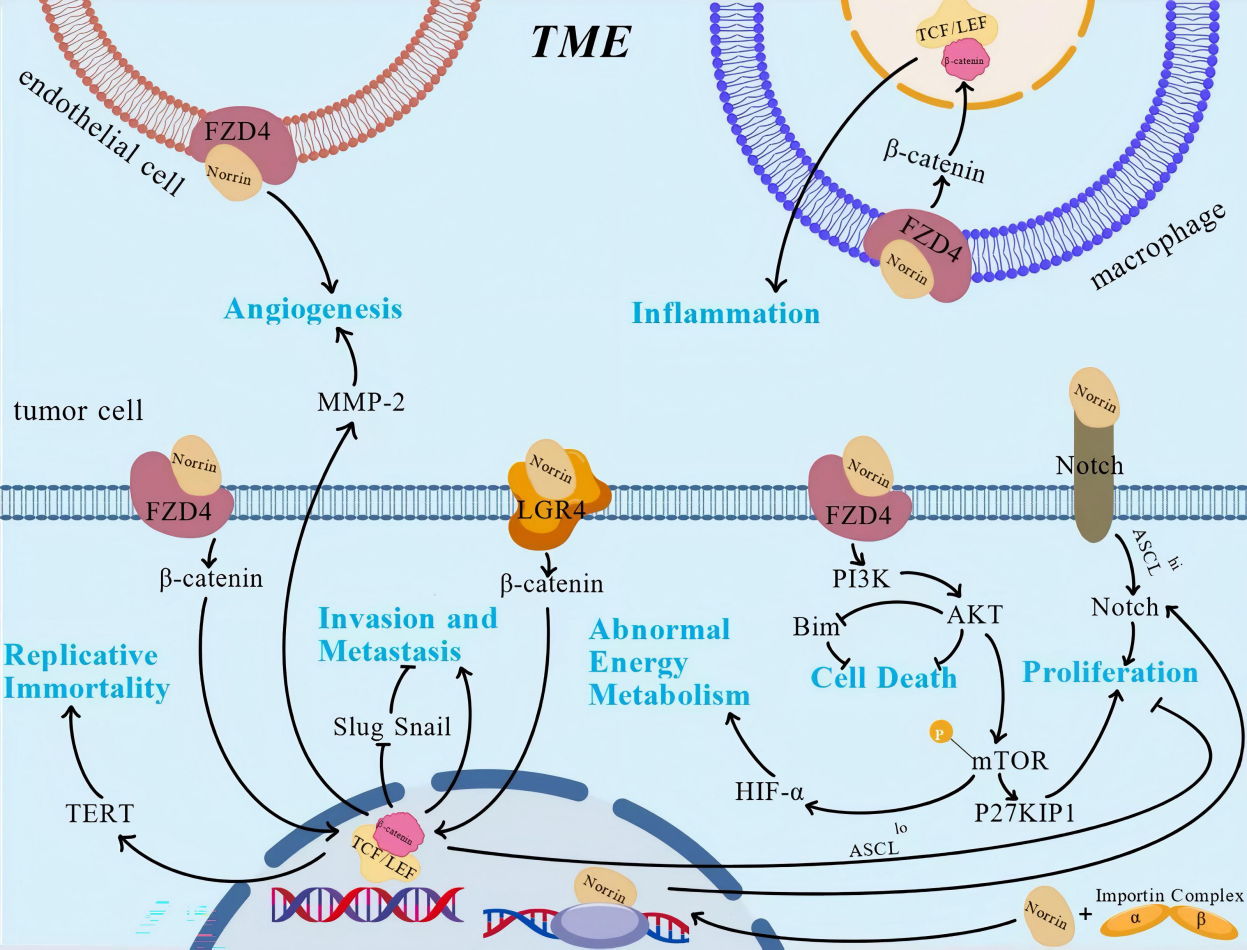
The role and regulatory mechanism of Norrin. Norrin regulates various characteristics of tumors through multiple pathways. This figure illustrates the regulatory mechanisms that have been studied relatively clearly, against the background of the tumor microenvironment, but it lacks the depiction of some mechanisms that have not been reported. This figure was created using BioGDP.com (33).

Accumulating evidence suggests that Norrin influences multiple hallmarks of cancer through diverse mechanisms, including sustaining proliferative signaling, promoting genomic instability, circumventing growth suppression, conferring resistance to apoptosis, enabling replicative immortality, stimulating angiogenesis, facilitating invasion/metastasis, metabolic reprogramming, immune evasion, and tumor microenvironment (TME) modulation (**Figure 4**). Acumulative evidences establish Norrin as a promising molecular target for novel antineoplastic therapeutics. Therefore, the cancer characteristics influenced by Norrin are highlighted and discussed ahead.

### 4.1 Effect of Norrin on the proliferation

Under normal physiological conditions, cell growth-promoting signals are strictly controlled during the modulation of cellular proliferation and cell cycle progression to maintain homeostasis and the normal functioning of cell tissues and the entire organism. The release and the regulation of these signals allow the autonomy and uncontrolled growth of cancer cells. Cell proliferation is regulated by various signaling pathways, such as proto-oncogenes and tumor suppressor genes (38).

Elevated *NDP* expression has been observed in multiple malignancies, including gastric and neurological cancers. The abundance of Norrin, which is encoded by the *NDP* gene, modulates cancer cell proliferation (41, 42). In gastric cancer (GC) cells, the cell proliferation ability decreases after Norrin is knocked out (16). Research has shown that Norrin deficiency can inhibit the phosphorylation of mTOR (16). As a central regulator of cellular growth and proliferation, mTOR is tightly regulated through signaling. Aberrant mTOR pathway activity is strongly associated with dysregulated cell proliferation, as demonstrated by extensive research (43). P27KIP1 is a regulator of cyclin-dependent kinases (CDKs) and has the potential to trigger cell cycle checkpoint activation (44). Loss of Norrin suppresses AKT pathway activity while increasing P27KIP1 expression, ultimately impairing gastric cancer cell proliferation (16, 45). Interestingly, the decrease in cell proliferation caused by Norrin deficiency can be restored by inducing the cytoplasmic activation of AKT using small molecules (16). Norrin also regulates brain cancer progression in humans, promoting tumor cell proliferation through multiple signaling mechanisms. In glioblastoma (GBM), the most common and aggressive malignant primary brain tumor, belongs to the family of astrocytic tumors, elevated *NDP* expression was significantly associated with prolonged survival, especially in patients with low ASCL1 expression levels (40). *In vitro* studies revealed that Norrin activates distinct signaling pathways in cells with different ASCL1 expression levels. In ASCL1^lo^ glioma stem cells (GSCs), Norrin suppressed proliferation through the FZD4-mediated Wnt/β-catenin cascade. Conversely, in ASCL1^hi^ GSCs, Norrin enhances tumor progression by activating Notch signaling in a Wnt-independent manner (40, 41). In proliferation and cell cycle assays conducted in previous studies, Norrin expanded the progenitor population and accelerated cycle progression, thereby increasing growth (40). After *NDP* gene knockout, the proliferation rate of GSCs decreased significantly (40).

### 4.2 Effect of Norrin on cell death

Apoptosis is an active and orderly process through which multicellular organisms eliminate abnormal or damaged cells. Apoptosis is crucial for maintaining internal environmental homeostasis. Conversely, malignant cells develop resistance to apoptosis through the dysregulation of Bcl-2 family members, inactivation of *TP53*, or reduced expression of apoptosis-promoting mediators (16, 38). Clear Cell Renal Cell Carcinoma (ccRCC) is the most common and aggressive subtype of renal cell carcinoma. The 5-year survival rate of patients with apoptosis-resistant metastatic ccRCC is just 5%, with curative outcomes virtually unattainable (46, 47).

Bim, is a BH3-only protein, also a crucial regulator of apoptosis, functions by interacting with the antiapoptotic Bcl-2 family members or directly triggering the pro-apoptotic effectors. Bim dysregulation is strongly implicated in tumorigenesis and cancer progression (48). Norrin mediates AKT pathway activation, which subsequently suppresses Bim expression, consequently attenuating apoptotic cell death (16). Furthermore, the AKT pathway intrinsically suppresses the apoptotic processes, and its constitutive activation represents a hallmark feature of numerous malignancies (49, 50). Therefore, it was speculated that Norrin is involved in the inhibition of cancer apoptosis.

### 4.3 Effect of Norrin on the invasion and metastasis

Most fatalities in oncology result from metastatic dissemination (51). Patients who develop drug resistance after chemotherapy experience metastasis and cancer cell spread to nearby tissues (52, 53). The metastatic potential of tumors fundamentally depends on their acquired capacity for cellular migration and tissue invasion. This process is initiated when neoplastic cells dissociate from the primary tumor mass, traverse through extracellular matrices, and ultimately infiltrate the adjacent tissues or disseminate to distant organs. Cellular migration represents the critical first step in the development of full invasive ability (54). The Extracellular Matrix (ECM) is a non-cellular, three-dimensional network of macromolecules that provides essential structural and biochemical support to surrounding cells. Cancer cell invasion involves the proteolytic degradation of the basement membrane and ECM components, which enables cancer cell penetration into neighboring tissues (54).

Norrin is essential for cancer cell invasion (16) and serves as an LGR4 agonist in malignant cells, stimulating Wnt/β-catenin signaling to drive metastatic behavior through enhanced motility and tissue infiltration, thereby facilitating tumor advancement (55). In gastric cancer cells, knockout of Norrin significantly decreases invasion ability (16). In pulmonary carcinoma models, elevated Norrin expression induced Wnt pathway activation, markedly enhancing the migratory and invasive capacities of the A549 and H1299 cell lines (18).

Epithelial-mesenchymal transition (EMT) is a key mechanism associated with tumor cell migration and invasion. In this process, epithelial cells undergo a defined transformation to adopt mesenchymal properties, and this is a critical step that facilitates tumor metastasis (56, 57). Moreover, the EMT process is associated with the drug resistance and stem cell characteristics of tumor cells (18, 38). A characteristic of EMT is the downregulation of E-cadherin, which is a critical mediator of epithelial cell adhesion (38, 58). Snail and Slug, two key transcriptional regulators involved in EMT, suppress E-cadherin and the related genes by targeting the conserved E-box sequence (59). Upon Norrin knockdown, the SPC-induced reduction in E-cadherin expression is reversed and is accompanied by a decreased expression of the EMT regulators Snail and Slug, which ultimately suppresses tumor cell migration and invasion (18).

### 4.4 Effect of Norrin on the replicative immortality and DNA repair

Unlimited replicative potential is a hallmark feature of cancer cells, and malignant cells achieve unlimited proliferation through telomere maintenance mechanisms (TMMs), evading replicative senescence and sustaining continuous cell division (60). Alternative Lengthening of Telomeres (ALT) is a telomere-maintenance mechanism used by some cancer cells to achieve immortality (61). Replicative immortality is achieved mainly through the telomerase and alternative lengthening of telomeres (ALT) pathways (62). DNA lesions represent a major source of genomic instability in cells (63). Telomerase plays a canonical role in telomere elongation and maintenance, while also performing extratelomeric functions, including its participation in the DNA repair processes (38, 64). TERT interacts with the key signaling components, including cMYC, NF-κB, and BRG1, enhancing tumor aggressiveness (62, 65–68). Therefore, interventions targeting DNA damage and DNA damage repair mechanisms could provide novel ideas for cancer treatment (69).

Norrin has never been shown to directly modulate telomere maintenance or telomerase function in cancer cells so far. Nevertheless, existing evidence suggests Norrin’s role in mediating Wnt/β-catenin pathway transduction (34, 70, 71). Research on colorectal cancer has demonstrated that Wnt pathway activation increases TERT expression, subsequently increasing the telomerase activity and preserving telomere length (72, 73). Therefore, we can analogical think that Norrin may affect TERT by activating Wnt/β-catenin pathway in some cases, however, no studies are available to provide direct evidence.

Genomic instability is an important characteristic of cancer, and uncontrolled proliferation of malignant cells promotes genomic instability, resulting in cumulative damage to key cell cycle regulators and tumor suppressors, which drives tumorigenesis and cancer progression (74–77). The role of p53, encoded by the *TP53* gene, as a critical growth inhibitor in malignant cells is well known. This tumor suppressor gene has the highest mutation frequency among all cancer-related genes and serves as a key oncogenic driver in multiple tumor pathologies (78). Cancer patients with mutations in the *TP53* gene have a poor prognosis (79, 80). The precise molecular mechanisms connecting Norrin signaling to genomic instability in malignancies remain to be elucidated to date. However, researchers have found, through STRING analysis, a protein-protein network interaction between Norrin and *TP53* (81). It was, therefore, speculated that Norrin may affect genome instability through *TP53* expression. However, some studies have demonstrated that the knockout of *NDP* can increase the mutation frequency of EC genes and contribute to the creation of the TME (19).

### 4.5 Effect of Norrin on tumor metabolism

The remodeling of cellular metabolism is a defining feature of aggressive cancers (82–84). In oxygenated environments, non-transformed cells predominantly utilize oxidative phosphorylation as their principal metabolic pathway for ATP generation. However, under anaerobic conditions, a relatively abnormal energy metabolic process called glycolysis may occur. Cancer cells, on the other hand, exhibit metabolic flexibility and favor glycolysis over oxidative phosphorylation even under normoxic conditions, and this is a hallmark of tumor metabolism known as aerobic glycolysis (85).

The AKT pathway has been established as a critical modulator of metabolic regulation in mammalian cells (86–88). After the AKT pathway is activated, phosphorylation and activation of mTOR occur, which promotes the expression of HIF-α, a key regulatory factor of glycolysis (89). HIF-α binds to glycolytic gene promoters, activating their transcription and upregulating the glycolysis-related pathways (89). Studies have indicated that Norrin stimulates the AKT/mTOR cascade (16). Hence, Norrin could be a key modulator of metabolic pathways in cancer.

### 4.6 Effect of Norrin in tumor microenvironments

Carcinogenesis is intricately linked to the alterations in the TME. The tumor microenvironment harbors cancer stem cells and bioactive factors that promote tumorigenesis by providing nutrients and mitogenic signaling, and through circulatory and lymphatic networks, the TME mediates the intercellular communication that orchestrates carcinogenesis and tumor progression (90–92). Traditional cancer treatment is based on targeting the tumor cells; however, given the critical function of the TME, analyzing the molecular and cellular dynamics of the TME during tumor development and discovering the potential therapeutic targets has gained significant attention in cancer research (93). TME drives the key oncogenic processes of angiogenesis, inflammation, immune evasion, and neural network integration. This report describes the effect of Norrin on these cancer hallmarks to explore the associations between Norrin and the TME (91, 94–96).

#### 4.6.1 Effect of Norrin on the angiogenesis

Tumor-associated angiogenesis is widely recognized as a key facilitator of malignant progression because it enables sustained proliferation and clonal evolution. The *NDP* gene product Norrin is a recognized regulator of vascular development. Clinical investigations have revealed that tumor-derived Norrin in colorectal carcinoma actively modulates endothelial cell behavior, with its presence observed alongside increased motility and the progression of pathological angiogenesis (97). Emerging evidence suggests that Norrin activates the Wnt/β-catenin signaling cascade, thereby upregulating MMP-2 expression (97). MMP-2 can hydrolyze type IV collagen and other connective tissue substrates and stimulate endothelial cell motility, promoting endothelial cell invasion of blood vessels through the basement membrane (17). Notably, colorectal cancer microenvironments generate Norrin, whereas local endothelial cells express their complete signaling machinery, enabling the autocrine regulation of the tumor vasculature (17, 98). However, the effects of Norrin on vasculature are highly context-dependent. This is exemplified by its opposing roles in medulloblastoma (19). In medulloblastoma (MB) of the cerebellum, one kind of malignancy, activation of the Norrin/FZD4-mediated vascular regulatory signaling axis inhibited the initiation of MB in the Ptch ^+/–^ mouse model. Loss of Norrin increases the mutation frequency of the genes associated with endothelial cells, including Esm1 (endothelial cell-specific molecule 1), Plvap (plasmalemmal vesicle-associated protein), and Emcn (endomucin), which form a precancerous matrix that is characterized by vascular remodeling. This process establishes a pro-tumorigenic niche during MB initiation, facilitating the malignant transformation of premalignant lesions (19). Additionally, in a study on ovarian cancer, Norrin was shown to have an inhibitory effect on angiogenesis, but the mechanism remains unclear to date (99). Collectively, these findings demonstrate the context-dependent roles of Norrin in tumor angiogenesis across developmental stages and tissue types.

#### 4.6.2 Effect of Norrin on the inflammation

Inflammation has been described as the seventh hallmark of tumors (100). Under normal conditions, the body’s inflammatory response is a defensive reaction to the stimulation of various injury factors, and this helps maintain the body’s normal physiological functions (101, 102). A large amount of randomized controlled trial and case study suggests that chronic inflammatory diseases mediated by the immune system, which have not been effectively controlled for a long period, can increase the risk of specific malignant tumors (103, 104). Inflammatory bowel diseases (IBD), particularly ulcerative colitis and Crohn’s disease, are well-established risk factors for colorectal carcinogenesis. The risk of malignancy increases with prolonged disease duration, increased inflammatory activity, and increased severity of mucosal damage (104, 105). Tumor-associated inflammation encompasses the inflammatory processes triggered by tumor initiation and progression, which enhance the occurrence and development of tumors by recruiting and activating inflammatory cells, thereby helping the early tumors acquire their characteristic capabilities (106). These findings suggest that innate immune cells, in particular, exert functionally significant pro-tumor effects during cancer development (38, 107). The inflammatory response mediates the release of bioactive compounds into the neoplastic niche, facilitating the ability of various markers, including growth factors that maintain proliferative signaling, survival factors that limit cell death, pro-angiogenic factors that promote angiogenesis, and enzymes that modify the extracellular matrix and drive angiogenesis, invasion, and metastasis, as well as elicit signals that induce the occurrence of EMT and activate other marker-promoting programs (38, 107).

A study on medulloblastoma in mice reported that meningeal macrophages regulate the initiation of tumors by participating in the chemokine signaling of pre-tumor cells (108). It has also been reported that Norrin regulates the genes with inflammatory regulatory functions in meningeal macrophages (109, 110), maintains the activation of meningeal macrophages during the critical precancerous stage, and inhibits the chemokine signaling of pre-tumor cells, thereby inhibiting the initiation of medulloblastoma in mice. Norrin may, therefore, be related to the occurrence of inflammation.

#### 4.6.3 Effect of Norrin on the immune evasion

Numerous studies have demonstrated that cancer stem cells achieve immune evasion through various means. Immune evasion is important for tumor cells to circumvent immune-mediated recognition and removal (39, 111). The abnormal metabolism of tumors and the effect of Norrin on the metabolism of tumors have been described above. Extensive research has demonstrated that the metabolic reprogramming in tumors facilitates cancer immune evasion (112, 113). No direct mechanistic evidence is, however, available linking Norrin activity to the immune evasion processes in cancer.

In glioblastoma, Norrin contributes to glioblastoma stem cell maintenance by functionally engaging the Notch signaling cascade (38). LGR4, a receptor for Norrin, is involved in immune modulation within the tumor microenvironment. Activation of the RSPOs/LGR4/ERK/STAT3 pathway by LGR4 drives the M2 polarization of Tumor-Associated Macrophages (TAMs). As the most numerous leukocytes in this environment, TAMs are highly plastic cells existing along a spectrum from pro-immunogenic M1 to immunosuppressive M2 phenotypes, with the latter facilitating tumor immune evasion (55, 114).

Collectively, these findings suggest that Norrin may contribute to tumor immune evasion mechanisms.

#### 4.6.4 Effect of Norrin on the nerve connection

The nervous system is widely spread throughout the body. Under normal conditions, the nervous system exhibits its established roles in motor control and sensory processing, while beyond these roles, neural regulation of the stem cell niches constitutes an essential axis for controlling cellular behavior and preserving homeostatic balance across tissues. The nervous system also regulates the cancer phenotype in a similar way, usually through neural mechanisms parallel to those in normal tissues (115). The neural connection of cancer is a type of connection established between the cancer cells and the nervous system, and through long-range signaling mechanisms, the nervous system facilitates tumor initiation, progression, and metastatic dissemination (116). This linkage significantly contributes to tumor initiation, progression, dissemination, and patient pain perception (115, 117–119). Currently, no research has confirmed the specific impact of Norrin on the neural connections of tumors. Norrin is essential for retinal neuron growth during embryonic development (120), and tumorigenesis is similar to embryogenesis. The hallmark features of stem cells include their proliferative plasticity, which enables both self-maintenance and commitment to diverse cellular phenotypes. Embryonic stem cells can develop into various cells required for mammalian development (121), and tumor stem cells have also been proven to have similar capabilities (122). Thus, it was hypothesized that Norrin can influence tumor-associated neural connectivity as well.

#### 4.6.5 The context-dependency of the Norrin signaling pathway

As described above, Norrin participates in multiple signaling pathways, and it is important to note that the activation of these pathways is highly context-dependent. The Norrin-related pathways are influenced by factors such as tissue type and tumor microenvironment.

The activation of these pathways exits tissue or microenvironment specificity. A study on glioblastoma stem cells (GSCs) demonstrated that Norrin signals through the intact FZD4–TSPAN12–LRP5 receptor complex, which can be activated according to the expression level of GSC subtypes, specifically the expression level of ASCL1 (123). In colorectal cancer cells, however, Norrin signals through a simplified receptor complex consisting of FZD4 and LRP5 without TSPAN12. This simplified complex specifically activates the angiogenic branch of the Wnt/β-catenin pathway (17).

Currently, there is a lack of direct and conclusive experimental evidence to demonstrate that common factors in the tumor microenvironment, such as hypoxia or inflammatory cytokines, have a direct impact on Norrin expression. Further research in this area is warranted, as establishing a clear relationship between tumor microenvironment components and Norrin could reveal novel targets for combined therapeutic interventions.

### 4.7 Regulation of downstream signaling partners and post-translational modifications of Norrin

Above, we have discussed the role of Norrin and its upstream signaling pathways across various types of tumor cells. Here, we will supplement this by elaborating on the downstream signaling partners and post-translational modifications regulated by Norrin. This will help clarify the precise molecular mechanisms driving its oncogenic potential and contribute to a comprehensive understanding of Norrin’s signaling network.

First, we systematically outline the downstream signaling partners, identifying the FZD4–TSPAN12–LRP5/6 axis as the core downstream signaling pathway of Norrin. In cancer cells, Norrin’s downstream signaling partners exhibit dual specificity based on both “cancer type” and “subtype”. For instance, in glioblastoma, under low ASCL1 conditions, the FZD4–LRP5–TSPAN12 complex stimulates Wnt/β-catenin signaling. In contrast, under high ASCL1 conditions, Norrin activates Notch signaling by inducing the Notch intracellular domain (NICD), which represents a less predominant signaling axis (40, 41). Gastric cancer relies on a simplified FZD4 complex to signal through the PI3K/AKT pathway (16).

Then, regarding post-translational modifications, we specifically describe three key types that influence Norrin’s activity, including glycosylation, disulfide bond formation and polymerization/oligomerization (124, 125). Glycosylation, Norrin possesses a conserved N-linked glycosylation site near its N-terminus, where a glycan chain is attached to an asparagine residue. This glycosylation modification is believed to contribute to Norrin’s stability by protecting it from proteolytic degradation and may also play a role in its efficient secretion and signaling function (124). Disulfide Bond Formation, the amino acid sequence of Norrin contains seven conserved cystine residues that form an intricate network of intramolecular disulfide bonds. These disulfide bonds are essential for the proper three-dimensional folding of Norrin, its efficient secretion, and its binding to the receptor FZD4 and co-receptors LRP5/6. The formation of disulfide bonds ensures both the stability and bioactivity of Norrin (125). Polymerization/Oligomerization, Norrin can form homodimers or higher-order oligomers via intermolecular disulfide bonds. This oligomerization occurs following secretion or within the extracellular matrix. Although the monomeric form of Norrin has been shown to possess bioactivity, its oligomeric forms may represent a storage state or serve to modulate signaling strength and duration by increasing local concentration and stability (124). The above descriptions supplement the previously dispersed introductions to the pathway by providing a systematic and consolidated overview of Norrin’s downstream signaling and regulatory mechanisms.

### 4.8 Mechanisms of interaction between norrin and other potential oncogenic pathways

Beyond the mechanisms discussed above, we hypothesize that several other signaling pathways may also regulate Norrin expression, such as the Hippo-YAP, TGF-β, Notch, and Hedgehog pathways. Below, we outline the rationale for this hypothesis.

First, the Hippo-YAP pathway is an evolutionarily conserved kinase cascade whose core effectors, YAP and the transcriptional coactivator with PDZ-binding motif (TAZ), play pivotal roles in regulating organ size, cell proliferation, differentiation, apoptosis, and tissue regeneration (126). As established, Norrin exerts its biological functions through the Wnt/β-catenin signaling pathway. Meanwhile, TAZ has been shown to restrict Wnt/β-catenin signaling via direct cytoplasmic interaction with Dishevelled (DVL), leading to suppression of Wnt pathway activity (127). Based on this evidence, we hypothesize that TAZ may interact with Norrin-mediated signaling through its modulatory effect on the Wnt/β-catenin pathway (128). Next, regarding the TGF-β pathway, although there is no direct evidence indicating that TGF-β regulates Norrin expression or Norrin directly modulates TGF-β signaling, high levels of TGF-β in the eye have been observed to significantly reduce the activity of the Wnt/β-catenin signaling pathway (129). Therefore, we hypothesize that TGF-β may influence Norrin-mediated signaling through this indirect mechanism. Norrin has been shown to stimulate Notch signaling by inducing the Notch intracellular domain (NICD) in glioblastoma (40), the specific signaling mechanisms involved remain unclear, warranting further investigation in future studies. Finally, concerning the Hedgehog pathway, while direct evidence of interaction is similarly lacking, in Norrie disease, expression of the *NDP* gene is initiated in retinal progenitor cells in response to Hedgehog signaling, which induces Gli2 binding to the *NDP* promoter (130). Based on this finding, we hypothesize that a similar regulatory mechanism may operate in tumor cells. The hypotheses outlined above merit further investigation in future studies, as their validation could provide insights valuable for leveraging Norrin signaling in clinical cancer therapy.

Through the foregoing discussion, it becomes evident that Norrin exhibits dual roles in regulating multiple hallmarks of cancer. However, the key determinants underlying this functional duality, specifically, the factors that drive the switch between its pro-tumorigenic and anti-tumorigenic effects, have not yet been comprehensively or conclusively elucidated in the existing literature. Further investigation into Norrin’s context-dependent functions is warranted, likely in relation to specific tumor microenvironmental conditions. Elucidating the factors responsible for these divergent outcomes is critical for guiding the clinical translation of Norrin-related therapies.

## 5 Biomarker and potential therapeutic options

### 5.1 Norrin as a biomarker

Norrin is highly expressed across multiple tissues (**Table 2**) and is critical for preserving the normal physiological functions in the body. For example, in normal cells, Norrin primarily modulates angiogenesis and exerts neuroprotective effect, studies have shown that recombinant Norrin markedly enhances vascular endothelial cell proliferation, viability, migration, and angiogenic capacity (6). So, the overexpression of Norrin may overwhelm these normal regulatory mechanisms, thereby affecting angiogenesis and indicating an abnormal pathological state, which directly promotes the progression of cancer. Therefore, Norrin can serve as a biomarker of cancer. As evidenced by its involvement in conditions like diabetic retinopathy and retinal vascular occlusion, the Norrin signaling pathway is crucial for retinal vascular development. Consequently, its dysfunction represents a promising therapeutic target for retinal vascular diseases (131).

**Table 2:** Norrin overexpression in the different tissue types.

Tissue	Samples overexpressed/ total aamples tested	Percentage of samples overexpressed(%)
Breast	18/1104	1.63
Central nervous system	38/697	5.45
Cervix	5/307	1.63
Endometrium	37/602	6.15
Hematopoietic and lymphoid	21/221	9.5
Kidney	38/600	6.33
Large intestine	37/610	6.07
Liver	20/373	5.36
Lung	4/1019	0.39
Esophagus	56/125	44.8
Ovary	13/266	4.89
Pancreas	6/179	3.35
Skin	12/473	2.54
Soft tissue	12/263	4.56
Stomach	27/285	9.47
Thyroid	14/513	2.73
Upper aerodigestive tract	17/522	3.26
Urinary tract	47/408	11.52

(Data from https://cancer.sanger.ac.uk/cosmic).

Beyond cancer, Norrin has been implicated as a potential biomarker in other diseases. Its biological function is primarily mediated through the activation of the Wnt/β-catenin signaling pathway (40, 132, 133). Elevated Norrin expression frequently correlates with the hyperactivation of the Wnt pathway, demonstrating pathological dysregulation of this signaling cascade. In kidney development and disease, Wnt signaling is rapidly reactivated after adult kidney injury (134). These findings suggest that overexpression of Norrin may activate the Wnt/β-catenin signaling pathway. This indicates that overexpression of Norrin reflects abnormal development or repair processes.

Clinically, elevated Norrin expression may function as a biomarker for both disease initiation and progression under specific pathological conditions. For example, the elevated expression of Norrin in inner ear tissue is associated with auditory pathology, including sudden sensorineural hearing loss, which may indicate vascular or cochlear dysfunction (135). Elevated Norrin expression can, therefore, be used as a biomarker in disease monitoring and prognosis judgment.

### 5.2 Potential therapeutic options

The relevant roles of Norrin in cancers have been detailed above, including the relevant receptors, several pathways, and currently known mechanisms. The development of relevant therapeutic drugs using Norrin as a therapeutic target is, therefore, worthwhile. However, to date, limited research has focused on identifying the specific inhibitors of Norrin, and no marketed Norrin-targeting agents have been reported for clinical application. Next, we will introduce some potential drugs that have been developed as Norrin inhibitors, and describe their research progress and drug toxicity. Here we propose a new concept: IC_50_, which is the half inhibitory rate used to evaluate the toxicity of compounds to cancer cells or other types of cells. And we have compiled all the drugs mentioned in this review and summarized them in a table to make them clear and concise (**Table 3**).

**Table 3:** Potential therapeutic options.

Classification	Name	IC_50_	Reference
Norrin-targeted antibody	Nb 1C4 and Nb 2B10	/	(136)
limb development membrane protein 1	LMBR1L	/	(137)
loop diuretic	Ethacrynic	105.9 ± 32.5 μM	(18, 138, 139)
Wnt pathway	Dickkopf	/	(140, 141)
	SFRP	/	(142)
	WIF-1	/	(143)
	Nitazoxanide	11.07 µM	(144, 145)
	Wnt-C59	74 pM	(146, 147)
AKT pathway	Wortmannin	30 nM	(148–150)
	LY294002	10 μM	(149, 151)
	AZD5363	200 nM	(152, 153)
	MK-2206	AKT1:5 nM, AKT2:12 nM AKT3:65 nM	(154)
	KRX-0401	/	(155)
Notch pathway	DAPT	140 nM	(156, 157)
	LY411575	72 nM	(158, 159)

First, we focus on Norrin-targeted antibody therapy. The extracellular localization of Norrin and its complex cystine knot structure pose significant challenges for drug design, delivery, and antibody targeting, representing a core bottleneck in advancing Norrin-based therapies. The intricate cystine knot structure has been clearly defined (130), consisting of two main components: a signal peptide at the protein’s N-terminus that guides its localization, and a region containing the canonical motif of six cystines that form the cystine knot. Nanobodies may serve as promising candidates for the therapeutic intervention of diseases associated with dysregulated Norrin signaling. Based on the structure of Norrin, researchers have now screened and identified two relevant Norrin-targeting nanobodies: Nb 1C4 and Nb 2B10, using flow cytometry, the binding affinity of selected nanobodies to the Norrin–FZD4 complex was evaluated, providing quantitative insights into their specificity. Furthermore, the study employed luciferase reporter assays to assess the functional impact of these nanobodies on Wnt/β-catenin signaling, enabling a comprehensive evaluation of their potential as therapeutic modulators (136). LMBR1L, a limb development membrane protein 1, it also lacks relevant drug toxicity studies, and currently preclinical studies have yielded results from *in vitro* experiments (137). Experiments *in vitro* have shown that the deletion of LMBR1L leads to the abnormal activation of the Norrin/β-catenin signaling pathway through the reduction in the ubiquitination of FZD4 and an increase in the expression levels of the Norrin coreceptor LRP5 and p-GSK3β-Ser9, indicating that LMBR1L has a regulatory effect on Norrin (137). Ethacrynic, a loop diuretic (138) (IC_50_ = 105.9 ± 32.5 μM) (139), it suppresses Norrin expression through the module of *NDP* transcription and translation (18). Ethacrynic has ototoxicity and has been found to cause edema and cellular changes in vascular lines when used in high doses (160).

**Figure 5:**
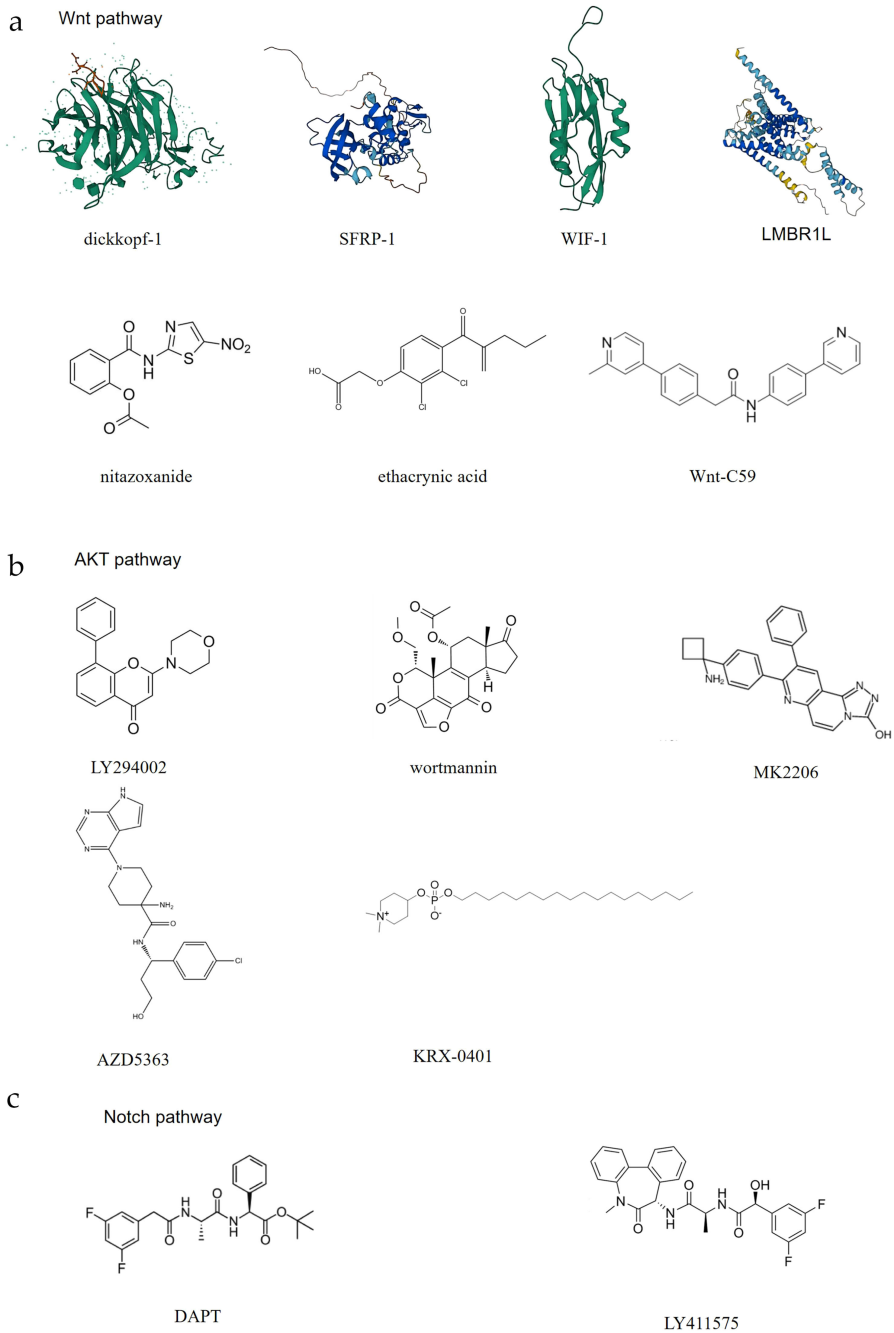
The potential inhibitors of Norrin. Here, the abovementioned compounds are classified and organized according to their mechanisms of action: **(A)** Exerts its effect through the Wnt pathway; **(B)** Exerts its effect through the AKT pathway; **(C)** Exerts its effect through the Notch pathway.

In addition, some pathway inhibitors may act on Norrin, and although there is no specific research regarding this, these compounds may be developed into novel targets for inhibiting Norrin based on their known mechanisms of action. Considering the effect of the existing pathway antagonists available in the market, Norrin is highly important for cancer patients. In this report, inhibitors are classified into three categories: antagonists of the Wnt pathway, the AKT pathway, and the Notch pathway (**Figure 5**).

When the Frizzled receptor (FZD) and LRP5/6 coreceptor form a complex upon Wnt ligand binding, the function of the destruction complex is inhibited, which results in cytoplasmic β-catenin stabilization and subsequent nuclear translocation. Following nuclear translocation, β-catenin associates with the TCF/LEF family transcription factors to assemble a transcriptional activation complex that drives the expression of Wnt target genes. These target genes primarily regulate different cellular processes, including proliferation, differentiation, and survival, thereby contributing to tumorigenesis and cancer progression (161). Currently, there are multiple drugs that act on different aspects of the Wnt pathway to inhibit Wnt signal transduction. The Dickkopf protein family, one kind of immunomo dulatory ligands (140) (Wnt pathway), can bind to LRP5, preventing its interaction with the Wnt-FZD complex and thereby antagonizing the canonical Wnt signaling pathway (141). The SFRP family, a FZD-related protein (Wnt pathway), can bind to the Wnt proteins through its N-terminal cystine-rich domain, inhibiting Wnt signal transduction (142). The secreted protein WIF-1 (Wnt pathway) binds to the Wnt proteins through its WIF domain, inhibiting Wnt signaling (143). Nitazoxanide, a clinically approved secreted mediators of Wnt/β-catenin signaling pathway (Wnt pathway) (IC_50_) = 11.07 µM) (144), induces β-catenin degradation, leading to the suppression of Wnt/β-catenin signaling (145). However, this effect is context-dependent. Wnt-C59, a Wnt signaling inhibitors (Wnt pathway) (IC_50_ = 74 pM) (146), reduces the interaction between β-catenin and NF-κB, acts as a Wnt signal inhibitor (147). Whether these Wnt pathway antagonists can also act on Norrin requires further research.

In addition to Wnt pathway modulation, several pharmacological agents have been identified as inhibitors of AKT pathway signaling. Wortmannin, a specific inhibitor of phosphatidylinositol-3-kinase (148) (AKT pathway) (IC_50_ = 30 nM) (149), can inhibit the activity of PI3K, thereby blocking the activation of the AKT pathway (150). LY294002, a PI3K inhibitor (AKT pathway) (IC_50_ = 10 μM) (149), also acts through PI3K. This compound binds the PI3K ATP-binding pocket, thereby suppressing AKT pathway activation (151). AZD5363, a novel, selective ATP-competitive pan-AKT kinase inhibitor (AKT pathway) ((IC_50_ = 200 nM) (152), has also been developed as an oral drug and can inhibit the three isoforms of AKT (153). MK-2206, an allosteric AKT inhibitor that inhibits AKT1, AKT2, and AKT3 (AKT pathway) (AKT1 [IC_50_ = 5 nM], AKT2 [IC_50_ = 12 nM] and AKT3 [IC_50_ = 65 nM]) (154), also has a similar effect (154). KRX-0401, an alkylphospholipid, is known as the first allosteri AKT inhibitor to enter clinical development and is mechanistically characterized as a PH-domain dependent inhibitor (AKT pathway) (155).

DAPT (IC_50_ = 140 nM) (156) and LY411575 (IC_50_ = 72nM) (158) are γ-secretase inhibitors (Notch pathway) (157, 159). The effects of these known pathway inhibitors on unknown targets may present both opportunities and risks, and by combining computational prediction and experimental validation, their effects on Norrin can be studied, while their clinical potential can be systematically explored to accelerate drug discovery and expand therapeutic indications. However, safety and specificity studies need to be conducted.

All potential drugs targeting Norrin mentioned earlier are currently at the research stage, and clinical trials have not yet commenced. In addition to the above compounds, despite preclinical evidence that Norrin-targeting antibodies remain unexplored in clinical cancer studies, monoclonal antibodies against Norrin are currently being used in basic research (17). Future investigations should explore Norrin’s therapeutic potential in clinical disease management.

## 6 Conclusions and perspectives

In this study, Norrin’s clinical applicability as a cancer biomarker as well as a therapeutic target is discussed, reviewing its mechanistic contributions to tumor biology, which involves cancer cell proliferation, death, migration and invasion, replicative immortality, metabolism, the microenvironment, angiogenesis, the inflammatory response, immune escape, and neural connections. Norrin is significantly overexpressed across multiple cancer types, with substantial clinical and experimental evidence linking elevated Norrin levels to tumor initiation and progression. However, to date, the effects of Norrin on several hallmarks of cancer, such as its context-specific regulation within the tumor microenvironment as discussed above, have not been fully elucidated. Further research in these areas is warranted, as it may uncover novel targets for cancer intervention. Although the biological roles of Norrin in the tumor microenvironment are increasingly understood, the regulatory mechanisms governing its expression in tumors. Particularly how epigenetic regulation and post-translational modifications influence its secretion, folding, and receptor interactions. Remaining a “black box” requires urgent exploration. Elucidating these mechanisms is of critical importance. First, deciphering how epigenetic mechanisms such as DNA methylation and histone modifications control *NDP* gene expression in tumor or stromal cells will help clarify the cell-type specificity of Norrin expression and may reveal novel therapeutic targets. Second, in-depth investigation into the post-translational modifications of Norrin is essential. For instance, while the correct formation of its seven disulfide bonds is known to be indispensable for its activity (125), the detailed folding process and potential variations in glycosylation patterns remain largely unknown. Furthermore, could dysregulation of these processes be linked to tumor heterogeneity and therapy resistance? Addressing these knowledge gaps will not only enhance our understanding of the dual roles of Norrin in cancer but also establish a solid theoretical foundation for developing precision strategies targeting this pathway, such as stabilizing or disrupting its functional conformation. Further research in these areas is warranted, as it may uncover novel targets for cancer intervention.

The considerable therapeutic value of Norrin as a molecular target, therefore, warrants further investigation for precision cancer medicine applications, especially research on nanobodies. No specific Norrin inhibitor has been discovered to date. It is necessary to find compounds that can specifically inhibit Norrin. These putative Norrin inhibitors are crucial for revealing the exact mechanisms and functions of Norrin, and such studies will simultaneously deepen the understanding of oncogenic mechanisms while accelerating the translation of Norrin-directed therapeutics into clinical applications.
